# Physical Exercise and Diet: Regulation of Gut Microbiota to Prevent and Treat Metabolic Disorders to Maintain Health

**DOI:** 10.3390/nu15061539

**Published:** 2023-03-22

**Authors:** Li Zhang, Yuan Liu, Xinzhou Wang, Xin Zhang

**Affiliations:** 1 Department of Physical Education, China University of Mining and Technology, Beijing 100083, China; 2 Department of Food Science and Engineering, Ningbo University, Ningbo 315211, China

**Keywords:** physical activity, diet structure, gut microbiota, metabolic diseases

## Abstract

Each person’s body is host to a large number and variety of gut microbiota, which has been described as the second genome and plays an important role in the body’s metabolic process and is closely related to health. It is common knowledge that proper physical activity and the right diet structure can keep us healthy, and in recent years, researchers have found that this boost to health may be related to the gut microbiota. Past studies have reported that physical activity and diet can modulate the compositional structure of the gut microbiota and further influence the production of key metabolites of the gut microbiota, which can be an effective way to improve body metabolism and prevent and treat related metabolic diseases. In this review, we outline the role of physical activity and diet in regulating gut microbiota and the key role that gut microbiota plays in improving metabolic disorders. In addition, we highlight the regulation of gut microbiota through appropriate physical exercise and diet to improve body metabolism and prevent metabolic diseases, aiming to promote public health and provide a new approach to treating such diseases.

## 1. Introduction

Humans have been studying gut microbiota for decades, and according to known data, the gut of regular humans hosts more than 100 trillion microorganisms, almost equal to the sum of all cells in the body [1,2]. Of these large and diverse microorganisms, some have evolved over thousands of years to form a reciprocal symbiotic relationship with their hosts, and the collection of these microorganisms is called the gut microbiota [3,4]. Research on gut microbiota has continued to make tremendous breakthroughs in the past. Up to now, a large amount of experimental data has confirmed that gut microbiota is closely related to immune and metabolic functions and can also affect the brain through the brain–gut axis, improving cognitive function and treating depression [5,6,7,8]. Of course, a large part of the reason why gut microbiota has such a high research value is that gut microbiota is not static, and it will be affected by various factors and change, mainly including genetics, physical exercise, and diet, and this change will directly or indirectly affect the human health status, such as obesity and hyperlipidemia [9,10,11,12]. Therefore, regulating gut microbiota by appropriate means is of great importance for preventing and treating some diseases and is an effective way to maintain human health.

Metabolic diseases are various diseases caused by disorders of human metabolism, typically including type 2 diabetes caused by disorders of glucose metabolism, as well as hyperlipidemia caused by disorders of lipid metabolism; these diseases seriously endanger people’s health and bring a lot of trouble to everyday life [13,14,15]. In the past, scientists have made great efforts to prevent and treat the disease and achieved certain results, and they pointed out that, in addition to drug treatment, the body’s metabolism can also be effectively regulated through lifestyle changes, including a healthy diet and appropriate physical exercise, which are both recommended [16,17]. Combined with the proven fact that the gut microbiota is an essential player in human metabolic processes, we have strong evidence linking the regulation of the gut microbiota to the treatment of metabolic diseases [18,19]. In addition, it has been found that transplanting gut microbiota from normal humans can have a therapeutic effect on metabolic diseases [20,21]. This finding provides important support for the possibility of treating metabolic diseases by regulating gut microbiota, which provides a new avenue for treating and preventing metabolic diseases in the future.

Among the many factors that affect gut microbiota, diet has become a hot spot for research because of its advantages of convenience, safety, cheapness, and efficiency. In our daily life, diet is a part of everyone’s life. Different regions have different food cultures, so scientists have found that there are certain differences in the composition of people’s gut microbiota in different food cultures, and these reflect different health statuses, the most typical example being the healthy Mediterranean diet (MD) and the high-fat Western diet (WD) [22,23]. The study noted that MD subjects had significantly higher gut microbiota diversity than WD subjects, and the researchers found that MD subjects also had significantly higher levels of short-chain fatty acids (SCFAs) in their intestines than WD subjects and that SCFAs are important gut microbiota metabolites that are strongly associated with human health [24,25,26]. In addition, the prevalence of metabolic diseases was also relatively higher in the WD population [27]. Therefore, it is not difficult to think about the possible effects of gut microbiota on human metabolic and the beneficial regulatory effects of a healthy diet on the gut microbiota. Comparing the nutritional combination of the two, we find that MD contains more bioactive substances, such as dietary polysaccharides and polyphenols, which will undoubtedly benefit human health directly and, of course, also act on gut microbiota at the same time, playing a better role in the treatment and prevention of metabolic disorders [23].

As with healthy eating habits, proper physical activity is one of the most recommended ways for doctors to maintain health and prevent metabolic diseases. Past studies have confirmed that physical exercise can accelerate the human body’s metabolic rate, improve metabolic disorders, and enhance human cardiopulmonary function, effectively reducing the incidence of obesity, type 2 diabetes, and other metabolic diseases [28,29,30,31]. However, there is another huge impact of physical activity that is often overlooked: the regulation of gut microbiota. It has been demonstrated that physical exercise can indeed affect the diversity as well as the composition of the gut microbiota [32,33]. For example, in the experiment of Emmanuel et al. [33], after six weeks of intense physical exercise, the gut microbiota of the experimental mice was changed, showing an increase in the level of *Bacteroidetes* and a decrease in the level of *Firmicutes*. Interestingly, this effect from physical exercise promotes the production of SCFAs, further promoting the metabolism of the body [34]. This provides a new direction for the treatment and prevention of metabolic diseases.

Metabolic diseases afflict thousands of people worldwide and are a public health problem that has to be addressed in today’s society. Gut microbiota has significant research potential as a breakthrough to promote metabolism that can be regulated by diet and physical exercise. In this review, we outline the role of diet and physical activity in regulating gut microbiota. In addition, we highlight the regulation of gut microbiota and the prevention and treatment of metabolic disorders by performing appropriate exercise and dietary changes, intending to find a new therapeutic option for the future development of this field.

## 2. Physical Activity, Diet, and Gut Microbiota

### 2.1. Effect of Physical Exercise on Gut Microbiota

As one of the most common health items in daily life, physical exercise is one of the ways people use to maintain a good figure and keep their mood happy [35]. However, in places that are difficult to perceive, physical exercise regulates the gut microbiota and further maintains the health of the body.

Studies have confirmed that physical exercise can regulate gut microbiota through various mechanisms, including promoting neurotransmitter and hormone secretion, increasing intestinal transit, and releasing myokines [36]. In their experiments, researchers have found that physical exercise causes changes in the composition and structure of the gut microbiota. For example, Yukitoshi et al. [37] divided the participating volunteers into an inactive group (energy expenditure > 3 metabolic equivalents for <15 min/day) and an active group (energy expenditure > 3 metabolic equivalents for >15 min/day), and sequencing of their fecal flora after a period of time showed significant differences between *Bacillaceae* and *Fusobacteriaceae*. In several investigations, researchers have revealed differences in gut microbiota and its complex network of relationships between the two lifestyles of sedentary and exercise. The study noted that, compared to active individuals, sedentary humans have a reduced diversity of gut microbiota, a less complex network of relationships, and a lower competitiveness rating, and these changes will affect the release of a number of important metabolites and neurotransmitters, causing a range of health problems [38,39]. In animal experiments, a further breakthrough was made in the regulation of gut microbiota by exercise. Evans et al. [40] caused disruption of gut microbiota in experimental mice through a high-fat diet. They found that exercise was effective in restoring the disrupted gut microbiota in a subsequent exercise experiment, as demonstrated by a negative correlation between exercise and *Bacteroidetes/Firmicutes*. In another experiment, the results showed a significant increase in the content of SCFAs in the intestine of exercising mice compared to non-exercising mice, in addition to changes in the composition and structure of the gut microbiota [41].

Up to now, there have been relatively few studies on the effect of physical exercise on gut microbiota. Still, according to the available experimental data, it is easy to see that exercise has a particular regulatory impact on the composition and structure of gut microbiota, and this effect is beneficial to human health. Given the importance of gut microbiota in the human body, research in this area should receive sufficient attention.

### 2.2. Dietary Proteins

The study of the effect of diet on gut microbiota has a more extended history than that of physical activity. Of course, as with many things, the impact of diet on gut microbiota has two sides: a healthy diet can help regulate gut microbiota and prevent various diseases. In contrast, an inappropriate diet can disrupt the homeostasis of gut microbiota and cause many health problems.

A high-fat diet, a representative of an unhealthy diet, increases the incidence of metabolic diseases, such as obesity and hyperlipidemia [42,43]. In subsequent studies, scientists have found that a high-fat diet can cause significant disruptions to the homeostasis of the gut microbiota and suggest that such alterations may be one of the causes of the increased incidence of certain diseases [44,45,46]. In the experiments by Velázquez et al. [47], the structure of the gut microbiota of the experimental rats was significantly altered by prolonged high-fat feeding, as evidenced by an increase in the abundance of *Ruminococcus*, *Dorea*, *Coprococcus,* and *Adercreutzia*, as well as a decrease in the relative abundance of *Anaeroplase* and *Turicibacter*, and the rats also showed signs of obesity and insulin resistance. Moreover, in addition to structural changes in the composition of the gut microbiota, metabolites associated with the gut microbiota have also changed. In one experiment, deoxycholic acid (DCA) levels were significantly elevated in high-fat-fed mice, and excessive levels of DCA would damage DNA and also disrupt the integrity of the intestinal epithelium, which is detrimental to health. Researchers believe that a high-fat diet disrupts the homeostasis of the composition structure of the gut microbiota, and this adverse effect leads to abnormal metabolism of bile acids in the intestine by the gut microbiota causing this phenomenon [45]. A high-fat diet can affect the homeostasis of the gut microbiota in several ways and can be harmful to human health.

In contrast to a high-fat diet, a balanced and healthy diet is effective in maintaining the homeostasis of gut microbiota, promoting the growth of beneficial bacteria, and suppressing harmful bacteria to ensure the individual’s health. The most typical example is the conscious intake of bioactive substances, such as tea polyphenols (TPs) and dietary polysaccharides, commonly found in daily life [48,49,50]. TPs are derived from tea leaves, and we can easily obtain them by drinking tea. The results of Liu et al. [51], through the establishment of an in vitro human intestinal model, showed that TP could effectively inhibit the growth of harmful bacteria, such as *Enterobacteriaceae*, *Bilophila*, and promote the growth of beneficial bacteria, such as *Bacteroides*, *Bifidobacterium*. In addition, another experiment also showed that tea consumption could promote elevated levels of SCFAs in the intestine [52]. Dietary polysaccharides are another biologically active substance that is readily available in daily life. They are found in vegetables and fruits and have a significant and beneficial effect on gut microbiota. The gut microbiota has carbohydrate-active enzymes (CAZymes), which allow them to utilize polysaccharides that the body cannot digest. These polysaccharides provide important energy to the flora and are further metabolized by the gut microbiota into important metabolite products that play an essential role in maintaining health [53,54]. The regulation of the gut microbiota by polysaccharides has a large experimental basis. For example, there was a significant increase in the abundance of *Lactobacillus* and *Bifidobacterium* in the intestine with the intervention of Coix polysaccharides; and, with the intervention of purple potato polysaccharides, it increased the abundance of *Oscillospira* and *Lachnospiraceae* and decreased the abundance of harmful bacteria *Sutterella* [55,56]. In addition, the intervention of dietary polysaccharides is accompanied by an increase in the content of important metabolites of the gut microbiota, such as SCFAs, which may originate from any food in life, including some healthy herbs [57,58,59].

**Figure 1:**
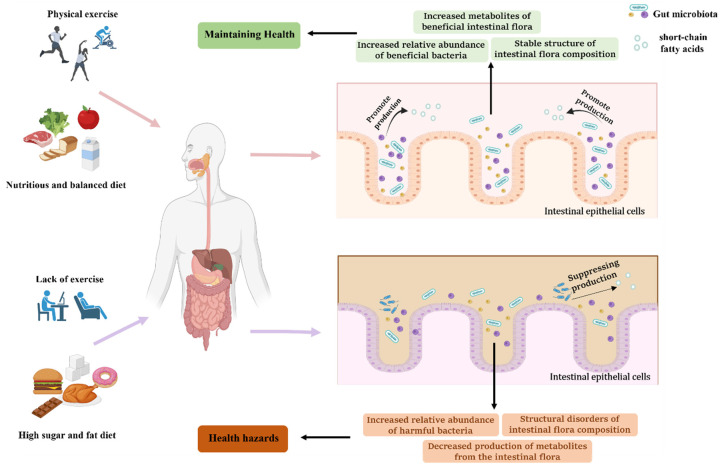
Effect of physical activity and diet on gut microbiota.

The effects of physical exercise and diet on gut microbiota are shown in Figure 1. Given the importance of gut microbiota for human health, dietary regulation of gut microbiota has great research value. Research on the prevention of metabolic diseases through dietary regulation of gut microbiota has made significant breakthroughs in recent years, and combined with daily physical exercise, this may become an effective way to reduce the incidence of metabolic diseases.

## 3. The Role of Gut Microbiota in Metabolic Disorders

### 3.1. Metabolic Disorders and Metabolic Diseases

People experience diseases throughout their lives. In our daily life, metabolic diseases, such as diabetes, obesity, and hyperlipidemia, have a very high incidence. Most of these diseases are caused by abnormalities in our own metabolic functions. This is a public health problem that we have to face. We need to understand these diseases and then prevent and treat them effectively.

Take the most typical example of diabetes mellitus, a metabolic disease caused by a disorder of glucose metabolism, which can be subdivided into type 1 diabetes and type 2 diabetes. Type 1 diabetes is caused by the destruction of the β cells of the pancreas and accounts for only 5–10% of people with diabetes, while more people have type 2 diabetes [60]. According to available reports, the development of type 2 diabetes can be summarized in two main points: insulin resistance and the destruction of pancreatic beta-cell function, resulting in the production of insulin that does not meet the body’s needs [13,60,61]. Statistics show that, as of 2017, the number of people living with type 2 diabetes worldwide has reached a frightening 462 million, accounting for 6.28% of the world’s population, and more than one million people lose their lives each year due to diabetes alone, making it the ninth leading cause of death in the world. What is even more worrisome is that scientists predict that the prevalence of type 2 diabetes will continue to rise in the coming years and that the age of death will gradually increase at younger age [62,63]. As a global disease, there is an urgent need for more effective and accessible treatment and prevention options for type 2 diabetes.

Hyperlipidemia is another common disorder caused by a disruption of the body’s metabolism. When the metabolism of fats or related functions becomes abnormal, the lipids or lipoproteins in the blood increases, causing hyperlipidemia [64,65]. Hyperlipidemia lays a hidden danger for various cardiovascular diseases, which seriously endangers people’s safety. According to research, hyperlipidemia not only affects the electrophysiological response and contractile function of the heart, but it is also a key factor in causing damage to the heart muscle and triggering atherosclerosis, which makes patients with hyperlipidemia far more likely to suffer from cardiovascular diseases, such as coronary heart disease and cerebral infarction, than normal people [66,67,68,69].

The causative factors of metabolic diseases are complex and diverse, and even today, we still cannot give complete results, but after years of ongoing efforts by scientists, many breakthroughs have been made in this field of research. The predisposing factors for metabolic diseases can be succinctly summarized as incorrect dietary habits, lack of exercise, and genetics [70,71,72]. With the continuous development of society, high-sugar and high-fat diet patterns and sedentary lifestyles have gradually become mainstream, which is the main reason for the rising trend of metabolic diseases year by year. It is noteworthy that, in recent years, scientists have pointed out that there is a very strong link between gut microbiota and human metabolism, and the composition of gut microbiota can be regulated by the influence of external factors. Interestingly, physical activity and diet have been shown to be among the main factors regulating the gut microbiota. This finding makes it possible that influencing gut microbiota through purposeful physical activity and regular diet has the potential to prevent and treat metabolic diseases and may be a new therapeutic option in the future.

### 3.2. The Close Association between Gut Microbiota and Metabolic Disorders

To prevent metabolic diseases, the most common thing we hear in our daily life is to exercise more and eat a proper diet. However, behind this “healthy life”, there is a key point that is often overlooked: the gut microbiota changes. According to several experimental data, it is indisputable that the composition of the gut microbiota of patients with metabolic diseases differs from that of normal people. As an example, in a study of the composition of the gut microbiota of patients with metabolic syndrome, He et al. [73] found that, compared to normal subjects, the structure of the gut microbiota composition was significantly disturbed, mainly by an increase in the relative fractions of *Firmicutes* and *Proteobacteria*, as well as a decrease in the relative fractions of *Ruminococcaceae* and *Bacteroidetes*. Similar results have been obtained in other animal and human studies. Researchers have shown that the composition of the gut microbiota of individuals with metabolic disorders is disturbed and loses its original diversity [74,75,76]. In addition, in another gut flora transplantation experiment, the important role of gut microbiota in the metabolic process was even more strongly demonstrated. Bidu et al. [77] induced metabolic syndrome by feeding mice high-sugar and high-fat food, and the results of their study showed that the transplantation of specific gut microbiota effectively intervened in the development of metabolic syndrome and played a role in preventing the metabolic disorders compared to the control group.

In addition to the most intuitive changes in the composition and structure of the gut microbiota, changes in the levels of important metabolites of the gut microbiota will also affect the metabolism of the organism. As representatives of the metabolites of the gut microbiota, SCFAs have been shown, in past studies, to play an extremely critical role in the metabolism process. According to the available studies, SCFAs can act as signaling factors to stimulate G-protein coupled receptor 41 (GPR41) and G-protein coupled receptor 43 (GPR43) in cells to activate AMP kinase, and, in addition, SCFAs can act as important energy donors to provide energy to intestinal epithelial cells and ensure the homeostasis of the intestinal environment [78,79,80,81]. By binding to GPR41 and GPR43 on cells, SCFAs can exert various beneficial health effects, including promoting metabolism. For example, Christiansen et al. [82] showed that SCFAs bound to GPR43 and GPR41 receptors on intestinal L cells, stimulating intestinal L cells and promoting the secretion of peptide-YY (PYY) and glucagon-like peptide-1 (GLP-1), play a large role in anti-diabetes and anti-obesity. To expand, PYY stimulates hypothalamic Neuropeptide Y (NPY) receptors, and the long-term activation of these receptors can play a role in suppressing appetite and protecting pancreatic β cells, which is important for the prevention of diabetes and obesity [83,84,85]. GLP-1 is secreted by intestinal L cells and plays a role in controlling blood glucose by enhancing insulin release and inhibiting glucagon secretion. In addition, GLP-1 delays gastric emptying and increases satiety, which is also important for the prevention and treatment of metabolic diseases [86,87,88]. In addition to SCFAs, which have been shown to contribute to human metabolism, another metabolite of the gut microbiota, secondary bile acids, has also been the subject of breakthroughs in recent years. Bile acids entering the intestine are dehydroxylated by a series of actions of the gut microbiota to produce secondary bile acids [89]. Studies have confirmed that both secondary and primary bile acids are signaling substances in the body and are involved in the metabolic processes of the organism. Bile acids can act on Takeda G-protein-coupled receptor 5 (TGR5) and farnesoid X (FXR), and the dual activation of TGR5 and FXR can effectively promote lipid and glucose metabolism and prevent the emergence of various metabolic diseases [90,91,92]. In addition, further studies have found that activation of TGR5 also improves the body’s sensitivity to insulin and that secondary bile acids also act on TGR5 on intestinal L cells to promote GLP-1 production, which plays a key role in promoting glucose metabolism, which has significant implications for the prevention of diabetes [93,94].

The metabolites of the gut microbiota are diverse, and in addition to beneficial metabolites, there are also harmful metabolites that can harm human health. Dysbiosis of the gut microbiota can cause elevated levels of these harmful metabolites, leading to a range of health problems, typically represented by lipopolysaccharides (LPS). LPS are bacterial surface glycolipids produced by Gram-negative bacteria [95]. Studies have demonstrated that LPS can bind to Toll-like receptor 4 (TLR-4) to induce the release of inflammatory factors and affect the development of immune cells, thereby causing a strong inflammatory response [96,97]. It is worth emphasizing that dysbiosis of the gut microbiota, one of the important components of the intestinal barrier, will not only increase the level of LPS, but will also lead to an increase in intestinal permeability, which makes it easier for LPS in the intestine to enter the bloodstream and seriously endanger health, including the effect on normal metabolism [95,98]. In the case of glucose metabolism, for example, researchers have found that LPS can cause or exacerbate diabetes by interfering with signaling pathways that impede insulin-related signaling, leading to a decrease in the body’s sensitivity to insulin [99,100].

**Figure 2:**
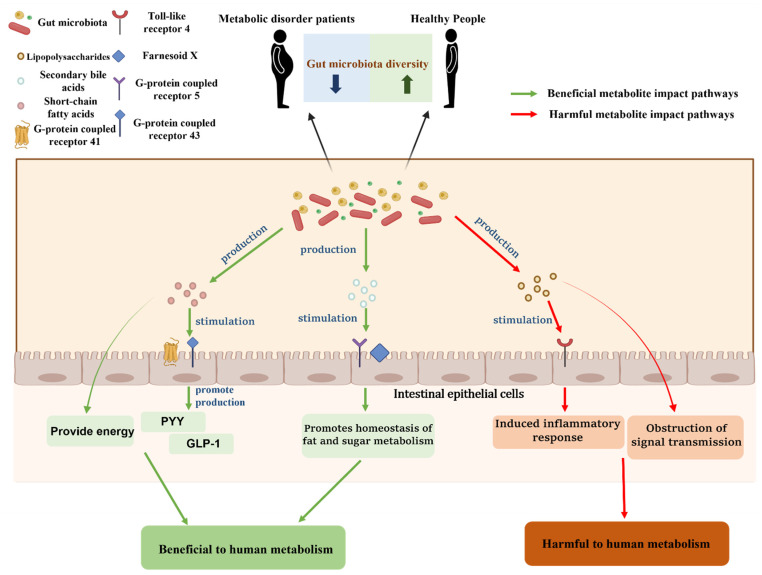
The link between gut microbiota and metabolic diseases.

In conclusion, the close link between gut microbiota and metabolic disorders, as shown in Figure 2, has been supported by numerous experimental data and is an indisputable fact. Thinking further, since gut microbiota can be regulated by external factors, based on the available data, we have the exact direction to improve metabolism by regulating gut microbiota and preventing and treating metabolic diseases. It can be summarized as preventing gut microbiota disorders, promoting the growth of beneficial bacteria and inhibiting the growth of harmful bacteria, promoting the production of metabolites of beneficial gut microbiota, and inhibiting the production of harmful metabolites. Given the important role of gut microbiota in human health, this field has great research potential. It can be boldly speculated that we can achieve the purpose of accurate regulation of gut microbiota through specific dietary arrangement and physical exercise, and through this purpose, we can promote the metabolism of the body, reduce the incidence of metabolic diseases, and solve health problems plaguing society, which is worth looking forward to in the future.

## 4. Physical Exercise and Diet, Together, Regulate Gut Microbiota to Prevent and Treat Metabolic Diseases

### 4.1. Treatment and Prevention of Type 2 Diabetes

In studying the human metabolism, modifying diet and increasing exercise are considered the most convenient and effective ways to promote metabolism and prevent disease. Type 2 diabetes should be taken seriously because of its extremely high incidence. It is important to know what benefits diet and exercise bring to our body’s glucose metabolism and how they can prevent the development of type 2 diabetes. We must then understand why diet and exercise are so important in the context of the link between gut microbiota and metabolism.

Let us first look at the improvement effect of physical activity on type 2 diabetes. According to past experiments, for type 2 diabetic patients, mild and moderate exercise significantly lowers blood glucose. For example, in the Jorge et al. [101] trial, patients participated in 60 min of aerobic physical training three times a week, and after 12 weeks, the patients’ fasting blood glucose levels decreased by an average of 12%; in the Kadoglou et al. [102] trial, patients underwent aerobic training for 24 weeks, four times a week for 60 min, and the patients’ fasting blood glucose levels decreased by an average of 24%, which was a significant decrease in blood glucose. The mystery of the ability of proper physical activity to lower blood sugar lies in its ability to promote the repair of damaged glucose homeostasis, as well as to improve the body’s sensitivity to insulin [103,104]. It is known that exercise increases the body’s need for energy, and the uptake of glucose by skeletal muscle in the exercise state is several times greater than in the calm state, but it is worth noting that strenuous exercise increases the release of glucagon and catecholamines, inhibiting the action of insulin, and one study also showed that elite athletes had higher levels of insulin resistance than sedentary subjects. Therefore, the choice of exercise intensity is also critical. To avoid high-intensity exercise, choose jogging, cycling, and other light and moderate exercise methos that are appropriate [103,105]. Combined with the ability of gut microbiota to control blood glucose as described above, it is easy to see that the exercise-induced reduction in blood glucose levels demonstrated in past experiments overlaps with the mechanism by which gut microbiota lowers blood glucose. Therefore, we can speculate that exercise plays a regulatory role on the gut microbiota and that this regulation contributes to glucose homeostasis in the organism and improves the metabolism of glucose to the organism. This conjecture has also seen breakthroughs in recent years of research. Pasini et al. [106] subjected 30 patients with type 2 diabetes to regular exercise for six months and showed that the patients’ previously disturbed gut microbiota was effectively restored, intestinal permeability was improved, and the diabetes was somewhat controlled. In addition, more convincing data were presented in another experiment. Torquati et al. [107] found that the composition structure of the gut microbiota of participating type 2 diabetic patients changed after eight weeks of low to moderate intensity aerobic exercise, as evidenced by an increase in the abundance of *Bifidobacterium* and butyrate-producing bacteria (*Enterococcus* spp., *Lachnospira eligens*), which were increased in abundance. The results showed that exercise had a regulatory effect on the gut microbiota of the patients, increased the production of SCFAs in the intestine, and that the metabolic capacity of the patients was significantly enhanced.

Genetic factors aside, an unhealthy diet is undoubtedly the main culprit of type 2 diabetes. In conjunction with past experiments, it is easy to see that the primary method of diet-induced secondary diabetes in experimental mice is feeding high-sugar, high-fat foods [108,109]. In turn, the best and easiest way to prevent type 2 diabetes is to modify our daily diet to include more fresh vegetables and fruits and to prevent excessive intake of sugar and lipids. The recommended dietary pattern is represented by a balanced meat and vegetable diet with a reasonable nutritional structure of MD. A statistical reports also showed that the incidence of type 2 diabetes is lower in people under MD, which plays an effective preventive role [110,111]. Diet is a key factor in the regulation of gut microbiota, and studies in recent years have pointed out that improvement of the dietary structure and the intake of appropriate bioactive ingredients will have a beneficial effect on the regulation of the gut microbiota and play a moderating, therapeutic role in addition to the prevention of related diseases. For example, dietary polyphenols commonly found in daily life are found in tea (green tea, oolong tea), vegetables (onions, broccoli), and fruits (lemons, grapes). Studies have shown that flavonoids and tannins in dietary polyphenols inhibit α-amylase and α-glucosidase, reducing glucose production from digestion; epigallocatechin-3-gallate (EGCG) increases glucose uptake by the body by activating AMP-activated protein kinase (AMPK) [112,113]. In addition, the researchers found that supplementation of dietary polyphenols in the daily diet also improved the composition of the gut microbiota, effectively lowering blood glucose levels and promoting the metabolism of glucose [114,115]. Dietary polysaccharides are another key “beneficial factor”. Li et al. [116] found that tea polysaccharides effectively restored the gut microbiota diversity of experimental rats that had been destroyed due to type 2 diabetes and promoted the production of related beneficial metabolites, and the final results showed a significant decrease in fasting blood glucose levels in type 2 diabetic rats under tea polysaccharide intervention. Similar findings were found for pumpkin polysaccharides, sea cucumber polysaccharides, pinecone polysaccharides, and other common foods [117,118,119]. The influence of diet on the gut microbiota is enormous, which indirectly affects the metabolism of glucosamine by the organism. In conclusion, if we want to prevent and treat type 2 diabetes effectively, our diet should be nutritionally balanced, avoid high sugar and fat, and consciously supplement with bioactive substances.

Today, the treatment of type 2 diabetes is still dominated by medications, which have side effects and are not affordable for everyone [120]. Fortunately, it is no coincidence that diet and physical activity have shown potential to prevent and treat type 2 diabetes, accompanied by improvements in gut microbiota structure and increased production of beneficial metabolites. Diet and physical activity are cheap and effective throughout everyone’s life. We can combine diet and exercise, and we can prevent type 2 diabetes with maximum efficiency by insisting on light to moderate exercise, such as jogging and cycling every day, ensuring three balanced meals a day, refusing high sugar and high fat, and purposefully regulating the gut microbiota in our bodies. Summing up various experimental data, the ability of exercise and diet to promote glucose metabolism is tremendous, but one point needs to be emphasized, controlling diet and performing exercise need long-term persistence.

### 4.2. Treatment and Prevention of Hyperlipidemia

Hyperlipidemia is a symptom caused by lipid metabolism dysfunction and is a major cause of cardiovascular disease. The question of effectively preventing and treating hyperlipidemia has been troubling scientists. Gut microbiota has received a lot of attention worldwide as a breakthrough in the study of metabolism in recent years. It has been found that diet and exercise can effectively regulate the gut microbiota related to lipid metabolism and provide a good effect in preventing and treating hyperlipidemia.

Physical exercise has been one of the effective ways to lower blood lipids. Studies have shown that exercise promotes the metabolism of high-density lipoprotein (HDL) in the blood and also has a positive effect on blood triglyceride (TG) and low-density lipoprotein cholesterol (LDL-C) levels, which are key factors in preventing hyperlipidemia [121,122]. A study of elderly patients with hyperlipidemia showed that, after six weeks of aerobic exercise, there was a significant improvement in total cholesterol (TCHO) and LDL-C levels, resulting in a better therapeutic effect [123]. The lipids in food enter the blood through a series of digestions in the body. A high-fat diet will directly increase the lipid content in the blood and cause hyperlipidemia, but relatively speaking, it is relatively easy for us to prevent it, and reducing the daily lipid intake can have an effective effect on lowering blood lipids [124]. Combining gut microbiota, diet, and exercise to lower blood lipids has again given new impetus to the research. The study points to differences in gut microbiota composition and reduced diversity in hyperlipidemic patients compared to normal subjects, for which the regulation of gut microbiota by diet and exercise is repeatedly highlighted above, which is a good start for more in-depth studies to follow [125]. Unfortunately, there are still very few animal and human studies on the treatment of hyperlipidemia through the regulation of gut microbiota by exercise, and there is still much room for research in this area. In contrast, diet-mediated alteration of gut microbiota has led to several breakthroughs in treating hyperlipidemia. According to the currently available experimental data, dietary polysaccharides, polyphenols, and flavonoids are hot spots for research and have yielded promising results, with typical cases bein shown in Table 1.

As with most metabolic diseases, the treatment of hyperlipidemia is currently focused on pharmacotherapy. Interestingly, studies in recent years have also revealed the linkage between some drugs and gut microbiota, such as simvastatin (SIM), a drug commonly used to treat hyperlipidemia, where scientists found that SIM effectively regulates gut microbiota associated with lipid metabolism and noted a correlation between this and its lipid-lowering ability [126]. Given the important role of gut microbiota in lipid metabolism, two key factors in regulating gut microbiota, exercise and diet, are of great research value. Looking ahead, it is not difficult to imagine that we may be able to prevent hyperlipidemia, reduce the incidence of hyperlipidemia in society, and consequently reduce the incidence of cardiovascular disease and address public health issues by developing a dedicated long-term exercise program and daily diet planning.

**Table 1:** Diet for hyperlipidemia through regulation of gut microbiota.

Diet Composition	Experimental Model	Symptoms	Treatment Methods	Affected Gut Microbiota	Experimental Results	Reference
TP	C57BL/6 mice (6 weeks old), high-fat diet feeding	Mice with hyperlipidemia	Daily intake of TP	Restores disturbed gut microbiota and affects the distribution of gut microbiota in the intestine	Hyperlipidemia symptoms have improved	[127]
*Auricularia auricula* polysaccharide	Sprague-Dawley rats (5 weeks old), high-fat diet feeding		Daily intake of *Auricularia auricula* polysaccharide	Increased relative abundance of *Lactobacillus*, *Roseburia,* and *Oscillibacter* and increased production of SCFAs in the intestine		[128]
Oat Flavonoids	C57BL/6N mice (5-week-old), high-fat diet feeding		Daily intake of Oat Flavonoids	The relative abundance of *Akkermansia* increased and the relative abundance of *Lachnoclostridium*, *Blautia*, and *Colidextribacter* decreased		[12]

## 5. Conclusions

Diet and physical activity, two important components of daily life, are of great importance for the maintenance of human health. Increasing the bioactive substances in the diet structure and doing some low to moderate intensity physical activity can effectively improve the composition structure of the gut microbiota and promote the production of beneficial metabolites in the gut microbiota. The gut microbiota and its metabolites can influence the metabolic process of human body through various ways, such as promoting the release of signaling factors and enhancing the intestinal immune barrier. The improvement of the composition of gut microbiota can effectively prevent the occurrence of metabolic disorders and thus treat type 2 diabetes, hyperlipidemia, and other metabolic diseases. In a long-term perspective, proper exercise and modification of the dietary structure have the advantage of being low cost and easy to implement. Therefore, the call for early planning of a proper diet and moderate exercise can effectively reduce the number of patients with metabolic disorders, which is a significant progress for the health of society.

## Data Availability

Not applicable.
